# Systemic Metabolism and Mitochondria in the Mechanism of Alzheimer’s Disease: Finding Potential Therapeutic Targets

**DOI:** 10.3390/ijms24098398

**Published:** 2023-05-07

**Authors:** Meiying Song, Xiang Fan

**Affiliations:** School of Basic Medical Sciences, Zhejiang Chinese Medical University, Hangzhou 310053, China

**Keywords:** Alzheimer’s disease, glucose metabolism, metal metabolism, insulin signaling, gut–brain axis, mitochondria

## Abstract

Elderly people over the age of 65 are those most likely to experience Alzheimer’s disease (AD), and aging and AD are associated with apparent metabolic alterations. Currently, there is no curative medication against AD and only several drugs have been approved by the FDA, but these drugs can only improve the symptoms of AD. Many preclinical and clinical trials have explored the impact of adjusting the whole-body and intracellular metabolism on the pathogenesis of AD. The most recent evidence suggests that mitochondria initiate an integrated stress response to environmental stress, which is beneficial for healthy aging and neuroprotection. There is also an increasing awareness of the differential risk and potential targeting strategies related to the metabolic level and microbiome. As the main participants in intracellular metabolism, mitochondrial bioenergetics, mitochondrial quality-control mechanisms, and mitochondria-linked inflammatory responses have been regarded as potential therapeutic targets for AD. This review summarizes and highlights these advances.

## 1. Introduction

Alzheimer’s disease (AD) is a neurodegenerative disorder and the most common and well-known form of dementia, accounting for 60–80% of all cases [1,2]. Pathologically, brain cells and nerves are damaged by the aggregation of proteins, mitochondrial dysfunction, and inflammation of the brain, forming neural disconnection, neural death and synaptic dysfunction in the brain [1,2].

Glycolysis and mitochondrial energy synthesis are intertwined in almost all cells and tissues, and together they support metabolism throughout the body. Although the brain is relatively small and isolated by the blood–brain barrier (BBB), the brain consumes approximately 20–25% of body oxygen and glucose [1,2]. Furthermore, the level of lipid metabolism in the brain is also the highest in the whole body, and its cells use part of the energy to replace the membrane lipid. The metabolic abnormality has been related to AD at the cellular level in the brain and across the entire body. Notably, those with type two diabetes (T2D) appear to be at a higher risk of developing AD [3]. Indeed, people 65 years of age or older have reduced insulin sensitivity [4,5,6]. Researchers have found common links between diabetes and AD based on epidemiology studies and investigations of clinical manifestations and mechanisms [7,8,9,10,11,12]. For instance, APP/PS1 mice exhibit decreased glucose tolerance and insulin sensitivity in AD models [13]. Streptozotocin, which has been shown to impair learning and memory and aggravate pathology in AD models, has also been shown to cause diabetes by disrupting pancreatic cell activity [14,15]. Although the precise molecular mechanism between diabetes and AD is unclear, these observations provide a potential AD treatment perspective.

Mitochondria are vital organelles that determine intracellular metabolic pathways, including Adenosine triphosphate (ATP) production via electron transport and oxidative phosphorylation (OXPHOS), the tricarboxylic acid (TCA) cycle, fatty acid β-oxidation, amino acid synthesis, calcium homeostasis, as well as iron metabolism. A high metabolic rate in mitochondria and the availability of oxygen both lead to the production of free radicals and, specifically, reactive oxygen species (ROS); thus, mitochondria are particularly vulnerable to ROS-mediated oxidative damage [16]. Mitochondria are one of the major producers of the ROS of brain cells in a physiological state, predominantly due to the leakage of electrons out of the electron transport chain [17]. In addition to mitochondria, ROS are mostly produced by the NADPH oxidase (NOX) in the cytosol [18,19,20]. Mitochondria generate and release pro-apoptotic molecules, particularly cytochrome c (Cyt c), which leads to programmed cell death [21,22]. Meanwhile, compromised mitochondria can also contribute to other forms of cell death, including necroptosis, ferroptosis, as well as pyroptosis [23]. Notably, abnormalities in the mitochondria’s electron transport chain activities have been seen in AD patients and model organisms. Thus, by focusing on mitochondrial function, homeostasis, and quality-control systems, age-related neurodegenerative disorders, including AD, have been successfully treated.

**Figure 1:**
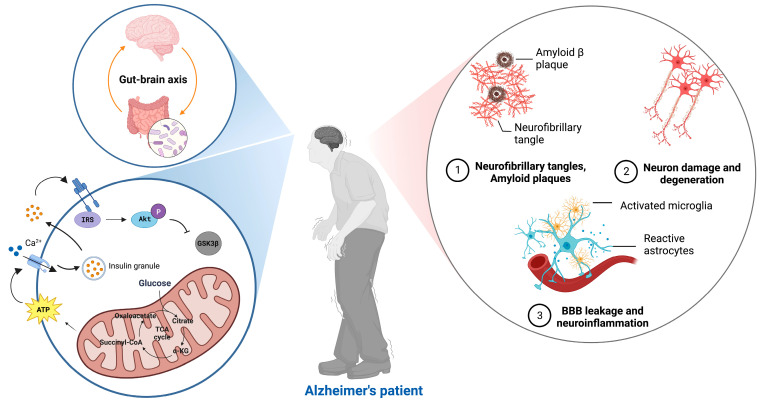
AD pathology related to metabolism and mitochondria. Alzheimer’s pathology is associated with aging, accompanied by impaired insulin signaling, unstable mitochondrial function, and an imbalance in energy metabolism.

This review will summarize advanced studies of metabolism, mitochondria and their association with AD pathology (Figure 1), and highlight the potential strategies for AD treatment.

## 2. Metabolic Alterations Occur during AD Pathology

The brain is an energy-demanding organ and relies heavily on efficient ATP production via glycolysis, the TCA cycle, and oxidative phosphorylation. The perturbation of glucose metabolism is a common feature of diabetes and AD. Targeting the whole-organism metabolism, we summarize and highlight the links between the glucose metabolism, metal ion metabolism, and insulin metabolism, and AD pathology. Furthermore, we discuss the interesting topics of metal ion metabolism and gut microbiota in AD mechanisms.

### 2.1. Glucose Metabolism and AD

Despite making up only 2% of the body’s entire mass, the brain consumes 20% of the body’s total glucose. The greatest energy-demanding neurons use glucose as their primary energy source. As a result, neuronal energy dyshomeostasis plays a role in neurodegenerative illness, which is defined by the destruction of neurons and a general decline in cognitive function.

It has been demonstrated in multiple clinical trials that energy dyshomeostasis appears to be associated with AD and AD-related dementias [24,25,26]. In biological systems, energy balance refers to the balance between energy intake and energy consumption over time [27]. The body’s overall and localized alterations in metabolism are reflected in the energetic state. Both weight loss and obesity are risk factors for developing AD [28,29,30]. Thus, clinical trials should consider BMI (Body Mass Index) and energy balance when designing their systems to understand the mechanism of energy imbalance associated with AD onset or progression [31,32].

Substrate- and tissue-specific metabolisms have been discovered using various AD models. In a pre-clinical study, diabetic rats with higher levels of amyloid exposure in the hippocampus had considerably lower calorie intakes, activity levels, and fat oxidation, while their carbohydrate oxidation and energy expenditure increased. This led to a negative energy balance and weight loss [33]. Cerebrospinal fluid (CSF) measurements using various indirect techniques have shown that, in patients with AD, lactate and pyruvate concentrations are higher, while succinate, fumarate, and glutamine concentrations are lower than in controls, revealing the damaged mitochondrial metabolism of glucose [34,35,36]. Impaired glucose metabolism (higher glycolysis) leads to high levels of lactic acid production, which is elevated in CSF due to the breakdown of the blood–brain barrier. In vivo imaging of brain atrophy can further confirm an abnormal cerebral glucose metabolism. Specifically, there are metabolic deficits in the precuneus, posterior cingulate, temporal-parietal, and hippocampal regions (consisting of discrete, bilateral, and symmetrical cortical areas), depending on the age of onset and progression of the disease [37,38,39,40,41,42]. The levels of glucose and associated metabolites in the brains of AD patients have been measured in case-control research, utilizing magnetic resonance spectroscopy. Elevated glucose to creatine ratios were detected, suggesting a reduced glucose metabolism in the brain [43]. On-resonance variable delay multiple pulse MRI was also used to examine the absorption of glucose in AD mice. Due to defective glucose transporters (GLUTs) across both the blood–brain and blood–CSF barriers, reduced D-glucose uptake in the cortex was seen, along with a decreased D-glucose uptake rate in the CSF [44].

In the early stages of the disease, BBB integrity is compromised, resulting in neurovascular dysfunction [45]. In one study, the BBB was disrupted, the amyloid-β protein (Aβ) buildup increased, and Aβ clearance was lowered in mice with overexpressed Aβ and GLUT1 deficiencies [46]. Aldolase, triosephosphate isomerase, glyceraldehyde-3-phosphate dehydrogenase (GAPDH), phosphoglycerate mutase 1, and enolase are among the glycolytic enzymes that are affected by oxidative alterations during AD [47], which results in a decreased glucose metabolism in the AD brain. Glycolytic product levels decrease with normal aging [48]. The expression of GLUT1 and GLUT3 is downregulated in AD, which reduces glucose transport and may impair cytoplasmic Ca^2+^ elimination [49]. The voltage-dependent anion channel (VDAC) on the outer mitochondrial membrane is where hexokinase binds. This interaction exhibits neuroprotective benefits by maintaining an outer membrane potential and closing the mitochondrial permeability transition pore (mPTP). Hexokinase levels are lower, and VDAC levels are higher in the AD brain [48].

In addition, glutamine/glutamate and fatty acid metabolisms are also the main subjects of brain metabolism. In AD patients and the old, the metabolic upregulation of fatty acid β-oxidation has been found to meet the additional energic requirement [50]. Furthermore, glutamate links amino acids to glucose metabolism through the TCA cycle (also known as the Krebs cycle), as aminotransferases regard glutamate as an ammonia donor, contributing to the production of 2-Oxoglutarate (2-OG) [51]. There is a reduction in glutamate levels in AD patients, accompanied by a decreased level of glutamine [52,53]. Furthermore, as glutamate binds to α-amino-3-hydroxy-5-methyl-4-isoxazolopropionic acid (AMPA) receptors and N-methyl-D-aspartate (NMDA) receptors, damaged NMDA receptor function results in Ca^2+^ dysregulation and decreased synaptic plasticity, which lead to cognition impairment in AD [54]. NMDA receptor-mediated excitatory glutamatergic neurotransmission is essential for synaptic plasticity and neuronal survival. However, too much NMDA activation results in excitotoxicity and cell death, providing evidence for a putative neurodegenerative pathway in AD [55,56].

### 2.2. Metal Metabolism and AD

Metal metabolism in this section mainly refers to the transport and accumulation of metals. For the brain to operate normally, iron and copper are necessary metals. They primarily work as co-factors in the synthesis of neurotransmitters, oxygen transport, synapses, and ATP. Iron regulatory proteins, such as ferritin, transferritin and transferrin receptor, hepcidin, ferroportin, and lactoferrin, are primarily responsible for controlling iron metabolism, whereas ceruloplasmin is mainly responsible for the metabolism of copper [57,58]. Advanced investigations have suggested that metal overload and the neurotoxicity that follows are factors in the pathophysiology of AD [59], with the most persuasive evidence coming from studies on copper and iron.

Free copper and iron ions in neurons trigger Fenton-like reactions that produce ROS and neuroinflammation [60]. According to a study from 2003, rabbits fed with high cholesterol chow and with excess copper in their drinking water had learning deficits after 10 weeks, suggesting the neurotoxic properties of free copper [61,62]. Notably, a lower level of ceruloplasmin ferroxidase activity in the blood of subjects with mild cognitive impairment, compared to controls, has been observed [63], while copper transport into the brain has been found to be aggravated by increased concentrations of circulating non-ceruloplasmin copper [64]. In addition, aceruloplasminemia, a rare disease characterized by iron deposition in the brain and cognitive decline, may also be caused by low ceruloplasmin levels [65].

Evidence has shown that increased labile copper may be associated with oxidative tissue damage in the brains of AD patients [66]. Notably, cuproptosis is a recently proposed mode of cell death, by which the aggregation of lipoylated proteins and proteotoxic stress, resulting from excessive copper accumulation, leads to cellular demise [67]. The role of cuproptosis in neurons and neurodegenerative diseases needs further exploration.

In addition, ferritin is the main way that the body stores iron. The level of ferritin in the CSF has been negatively associated with cognitive performance, which could also help to predict the rate of Mild Cognitive Impairment conversion to AD [68]. Recent studies have shown that iron levels in different parts of the brain are associated with AD pathology. For example, it was found that the iron concentration in the deep grey matter and neocortical regions was higher in patients with AD than in healthy control participants who underwent 3-T MRI [69]. Moreover, changes in iron levels over time in the temporal lobe have been associated with accelerated cognitive decline in individuals with AD [70]. In addition, it has been found that those who carry the ApoE4 allele have high levels of CSF ferritin [71]. Furthermore, iron and copper ions bind to the phosphorylated tau protein before intracellular tangles form, and metal ions influence aggregation [72]. The accumulation of these similar transition element cations in AD plaques, particularly copper, causes oxidative damage and speeds up the Aβ cascade, directly affecting neurodegeneration in mice models with severe neurodegeneration [73]. In addition, ferroptosis is the pathological process of AD that causes neuronal death [74], highlighting iron interactions and lipid dysmetabolism in AD pathogenesis.

In an in vitro study of SH-SY5Y cells overexpressing the Swedish mutant form of human β-amyloid precursor protein cells, iron treatment worsened oxidative stress damage and the production of Aβ1-40, and reduced cell viability and the mitochondrial membrane potential [75]. Although the precise mechanism of iron metabolism in AD pathology remains to be further explored, existing evidence suggests that it may be related to oxidative stress and Aβ deposition in the brain.

### 2.3. Insulin Signaling and AD

Insulin is a hormone that connects nutrients and metabolic homeostasis to systemic growth and development. Insulin is also regarded as an important participant in fertility and life span. In addition, this hormone also plays a major role in neuronal growth, connectivity, survival, and integral brain function. It has already been shown that AD and other neurodegenerative diseases are accompanied by peripheral and/or central insulin resistance. In AD pathology, elevated insulin levels can lead to extracellular regulatory kinase activation, which phosphorylates tau at Ser202, resulting in an increased number of neurofibrillary tangles [76,77]. Microtubules in the axons of neurons become unstable as a result. Insulin modifies Aβ intraneuronal concentration through the phosphoinositide 3-kinase (PI3K)/serine/threonine kinase (Akt, also known as protein kinase B or PKB)/glycogen synthase kinase 3 (GSK-3) pathway. Notably, insulin has anti-inflammatory properties as well [78]. Therefore, insulin can enhance emotions, behavior, cognition, learning, and memory capacities through its trophic and neuroprotective function in cerebral cells, protecting neurons from Aβ toxicity, oxidative stress, and apoptosis. Meanwhile, antidiabetic drugs are proven to have great potential in treating AD [79].

**Figure 2:**
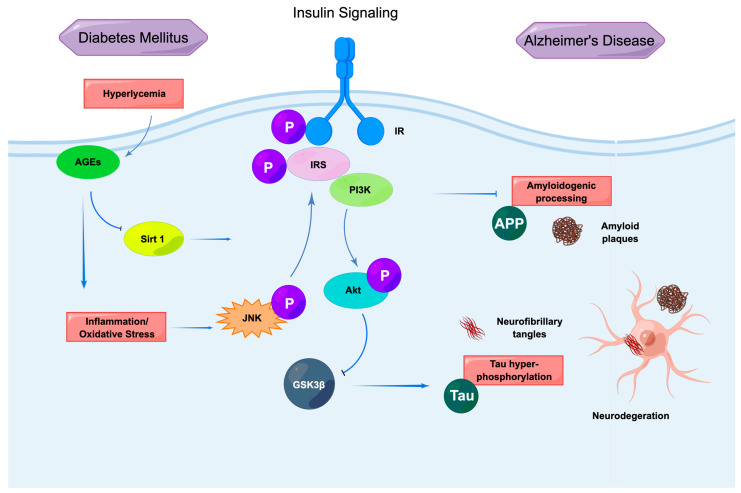
Insulin signaling is linked to T2D mellitus-related changes with AD. Insulin evokes the phosphorylation of IR and IRS, and activates Akt, ultimately resulting in improved glucose metabolism. Damaged insulin signaling directly or indirectly promotes the pathological progression of AD.

The c-Jun N-terminal kinase (JNK) pathway and the consequent phosphorylation of the insulin response substrate (IRS)-1/2 are two mechanisms shared by T2D and AD that contribute to insulin resistance and its subsequent dysmetabolic consequences (Figure 2).

IR in AD may be caused by peripheral impaired insulin signaling, one of the mechanisms shared by T2D and AD. In addition to insulin’s anabolic, mitogenic, and prohypertrophic effects, the RAS/MAPK cascade is associated with insulin’s mitogenic and prohypertrophic effects [80,81]. Notably, as a member of the MAPK, the JNK family has recently shown its important role in AD; this is not only due to the increased phosphorylated JNK expression in human postmortem brain samples and its positive co-localization with Aβ, but also due to its interference with the physiology of the neuronal insulin axis [82]. AD pathologic progress includes several stressors that are known to activate the JNK pathway, such as oxidative stress, Aβ accumulation, neurotrophic deprivation, and proinflammatory cytokines, such as tumor necrosis factor-alpha [83]. JNK activation, when triggered by free radicals or proinflammatory cytokines, could phosphorylate IRS 1, and then block the transduction signal produced by the insulin receptor [84]. IR induces the central depletion of neurotrophic factors and glucose-related energy dyshomeostasis, which makes neurons more vulnerable and amplifies JNK pathway activation. For insulin to have any physiological effects, tyrosine kinase activity receptors are required. Tyrosine autophosphorylation enables the gain-of-function of the receptors and the downstream phosphorylation of additional substrate proteins, known as IRSs 1 to 4 in mammals [85].

Numerous signaling partners can bind to the tyrosine phosphorylated IRS, and a specialized and compartmental pattern of physiological responses is determined by the tissue-specific expression and differential binding of these downstream signaling proteins [85]. Among them, PI3K has a significant role in insulin function, including the translocation of glucose transporter proteins, glycogen, lipid and protein synthesis, anti-lipolysis, and the control of hepatic gluconeogenesis [86]. PI3K works mainly via the activation of the Akt/PKB and the protein kinase C cascades [86]. Activated Akt then induces glycogen synthesis by inhibiting GSK3 protein via the mammalian target of the rapamycin (mTOR) and downstream elements [87]. Furthermore, Akt is beneficial for cell survival by inhibiting several pro-apoptotic factors, including the Bcl-xL/Bcl-2-associated death promoter, the forkhead box O (FoxO) transcription factors, and GSK-3 [88]. In addition, Akt phosphorylates and directly inhibits FoxO transcription factors in order to regulate metabolism and autophagy.

Insulin and insulin-like growth factors are crucial to the peripheral and central metabolism, as shown by the cellular abnormalities induced by the metabolic stress present in both T2D and metabolic syndrome. Targeting insulin resistance may be a possible strategy in the treatment of AD, although the underlying mechanisms of insulin involved in AD pathogenesis still need to be thoroughly established.

### 2.4. The Gut Microbiota and AD

AD was long believed to be a brain disorder without obvious connections to other distant tissues. However, this viewpoint has shifted over the past 20 years, starting with retrospective research in 1994. Sonnenberg et al. collated millions of medical records from the U.S. Department of Veterans Affairs and found that compared with normal people, patients with presenile dementia, AD and other neurodegenerative diseases had a significantly higher rate of experiencing colonic dysfunction [89]. This phenomenon suggests that gut dysfunction is strongly associated with the development of neurodegenerative diseases in the brain and related pathologies.

**Figure 3:**
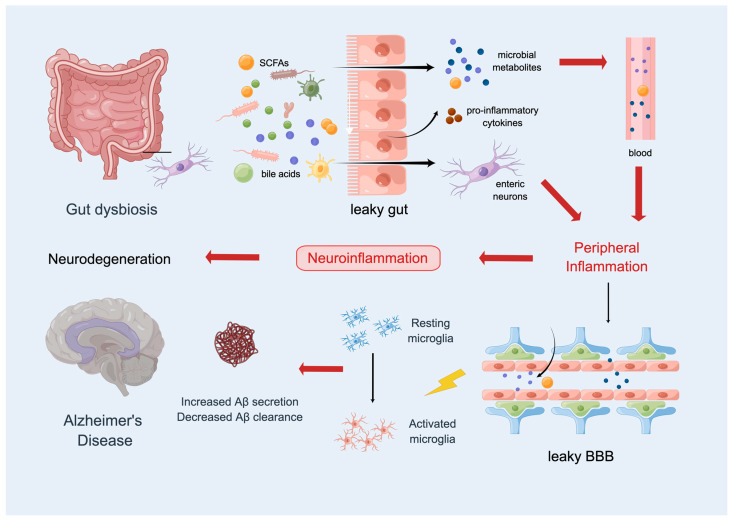
The gut–brain axis in AD pathology. A common link between metabolic dysregulation and AD is the dysbiosis of the gut. The gut–brain axis (the brain affects the gut via neuronal and hormonal signals, and the gut microbiome affects the brain) is a potential target for managing AD.

Additionally, Section 1 has shown how the pathogenic processes of AD are closely related to metabolic diseases, including obesity, metabolic syndrome, and diabetes, all of which are characterized by insulin resistance and a dysregulated glucose homeostasis [90]. A number of metabolites in the human body are strongly correlated with the human gut, and alterations in the density and/or makeup of the gut microbiome may result in metabolic diseases. More than 100 trillion bacteria, which encode >150 times as many genes as the human genome, live in the human gut [91,92,93]. Firmicutes and Bacteroides are the two most prevalent phyla in the guts of humans and mice, respectively [94,95]. Emerging evidence shows that the gut microbiota plays a vital role in the central nervous system in AD [96]; for example, it is involved in myelination, neuroinflammation, the modulation of BBB integrity, and a-synuclein accumulation, which are all aspects of neuronal activities [97]. The “gut–brain axis”, a collection of various neural networks that comprises the central nervous system, the autonomic and enteric nervous systems, as well as the peripheral nerves, mediates these ongoing connections between the brain and gut [98] (Figure 3). 

Emerging evidence regarding the function of the gut–brain axis and its influence on neurodegenerative disease has come from studies of the vagus nerve; this has been supported by an assessment of the incidence of dementia in patients with truncal vagotomy and concluding that vagotomy reduces the risk of developing dementia [99]. Not only does Aβ act as an initiator of neuroinflammation, but it has also been indicated that the peptide may be part of a response that is mounted by the innate immune system, which could mediate cytokine release and adaptive immune responses [100]. Intestinal inoculation with Aβ1-42 oligomers, and the subsequent spread of Aβ to the cholinergic neurons of the gut, cause dysfunction in specific gut regions; this ascends the vagus nerve to the brain, leading to cognitive deficits [101]. How Aβ spreads in the brain–gut axis and causes persistent dysfunction only in specific gut regions remains unknown. Notably, patients with AD have a lower ratio of Firmicutes to Bacteroidetes and a less diverse microbiome [102]. However, evaluations of the altered makeup of the gut microbiota have shown contradictory results in other AD investigations utilizing both humans and animals [97]. This discrepancy may be related to age, diet, and other factors. Additionally, changes in the gut microbiota and associated changes in the bacterial metabolism and metabolites may increase intestinal and BBB permeabilities. Short-chain fatty acids, SCFA, and other molecules derived from the gut microbiota accumulate in the brain due to increased permeabilities, causing inflammatory conditions in the microenvironment and laying the groundwork for the pathogenesis of neurodegenerative disorders such as Parkinson’s disease and AD [103]. Increased levels of the microbiota’s metabolites in the bloodstream, as well as humoral (such as pro-inflammatory cytokines) or cellular (like monocytes) effectors of peripheral immunity, may cause further pathogenic alterations. The function of microglia (brain-resident macrophages) and the imbalance of T-cells are influenced by the gut microbiota via the gut–brain axis [104,105]. For instance, SCFAs, a particular bacterial metabolite, influence glycemic regulation in various metabolic situations and are crucial to the metabolism of lipids and proteins [106]. The investigators found that SCFAs could promote microglial maturation, activation, and the generation of disease-associated microglia (DAM), a subset of central nervous system-resident macrophages found at sites of neurodegeneration; these may play a protective role in increasing plaque loads by helping to increase deposition and reduce clearance [107,108].

There is no doubt that the gut–brain axis is a vital integrated system that regulates metabolic balance and connects nerves and the gut, and that it could be a potential target for the prevention and treatment of AD. However, how the gut microbiome affects the systemic metabolism in the brain and circulation, and what the exact mechanisms involved in AD pathogenesis are, remains unclear. The ways in which dietary modifications and prebiotics affect the gut microbiota and neuronal energetics require further evaluation.

## 3. Mitochondria Function in AD Pathology

**Figure 4:**
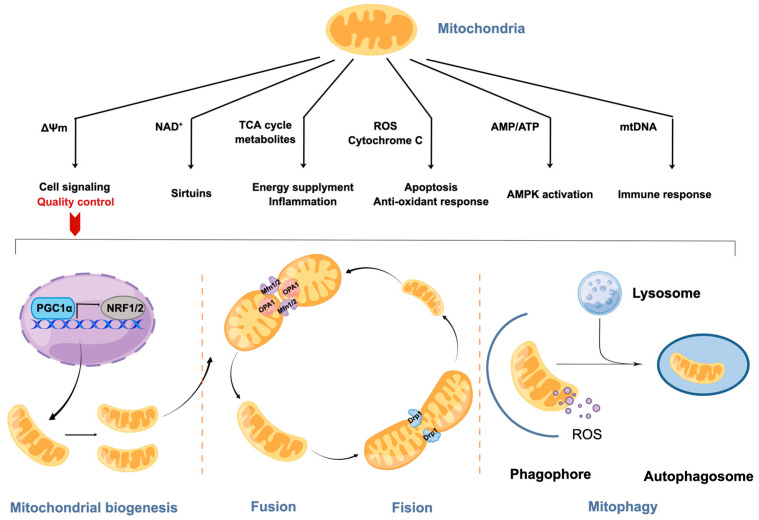
Mitochondrial intracellular signaling and mitochondrial quality control. Mitochondria not only provide energy but also work as signaling organelles. Mitochondria are dynamic, and several mechanisms work together to control the quality of mitochondria to ensure mitochondrial function.

Mitochondria play a crucial role in bioenergetics and metabolite homeostasis. Additionally, in the past two decades, it has also been indicated that mitochondria serve as the signaling hubs in which intra-cellular and extra-cellular signals, and communications with other cellular compartments, are orchestrated in order to achieve various functions under homeostatic and stressful conditions (Figure 4).

### 3.1. Mitochondria in Energy Production

The brain has various cell types, and each type has unique metabolic requirements and energy requirements. The most commonly studied energy supply organelle is mitochondria, where two biological processes, including the TCA cycle and OXPHOS, occur [109]; typically, neurons mainly depend on energy produced by mitochondria via OXPHOS [109]. OXPHOS is the most effective method of generating energy, generating 36 ATP molecules for every glucose molecule, instead of glycolysis’s 2 ATP molecules [110]. Three major nutrients in the body, including glucose, lipids and proteins, have different metabolic pathways; they all have the common intermediate metabolite acetyl-CoA, which is utilized by the TCA cycle to produce nicotinamide adenine dinucleotide (NADH) [111]. Furthermore, NADH is used as a substrate for OXPHOS to produce ATP and subsequently produce energy [112]. Notably, maintaining mitochondrial integrity is essential, since OXPHOS occurs at the inner mitochondrial membrane and the TCA cycle produces the components needed to power OXPHOS in the matrix. Moreover, neurons establish axonal and synaptic membrane potentials and sustain ionic gradients via mitochondrial ATP production. During OXPHOS, substrates that come from the TCA cycle, such as NADH or flavin adenine dinucleotide, donate electrons. The high-order assembly of respiratory chain complexes in the inner mitochondrial membrane, which is called the electron transport chain (ETC), promotes efficient electron transfer [113]. Subsequently, undergoing a series of redox reactions, adenosine diphosphate (ADP) is finally phosphorylated to produce ATP, CO_2_, and water. The ETC includes four protein complexes: Complex I (NADH dehydrogenase), Complex II (succinate dehydrogenase), Complex III (cytochrome c oxidoreductase), and Complex IV (cytochrome c oxidase) [114]. A protonmotive force (PMF) is created by electrons moving through the ETC, which encourages protons (H^+^) to move from the matrix to the intermembrane region. The PMF’s energy is used to phosphorylate ADP to ATP via ATP synthase (complex V) [115].

However, the steady Ca^2+^ levels in the mitochondrial matrix firmly regulate the activity of OXPHOS. Under pathological conditions, the mitochondrial metabolism is damaged due to excessive Ca^2+^ conversion by the endoplasmic reticulum (ER) or increased cytosolic Ca^2+^, which can contribute to oxidative stress, the prevention of mitochondrial ion exchange via the membrane, and the reduced production of ATP; all of these factors can lead to cell damage and even death [116]. It has been demonstrated that neurons in the AD brain exhibit Ca^2+^ dyshomeostasis and mitochondrial dysfunction. Mitochondria, the energy centers of the cell, are essential for cellular growth. Reduced ATP levels and mitochondrial function with increased ROS production frequently precede many diseases characteristic of Alzheimer’s [117,118]. Meanwhile, AD patients can also experience mitochondrial damage in peripheral tissues.

Since mitochondria are the site of TCA cycling and respiration and regulate ion transport, mitochondrial dysfunction is a major intracellular event that contributes to AD pathology. Mitochondrial abnormalities, such as a decreased respiratory capacity, increased mitochondrial fragmentation, and fragmented mitochondrial cristae, have been seen in AD brains prior to pathological Aβ plaque deposition [119,120]. Therefore, it is crucial to develop strategies that target mitochondrial function.

### 3.2. Mitochondria as Signaling Organelles

In the past few years, the ability of mitochondria to act as signaling organelles in order to regulate cellular fate has also been gradually revealed [121]. Mitochondria release ROS, metabolites and mitochondrial DNA (mtDNA) to communicate with the rest of the cell by changing their structures and motility and interacting with other subcellular organelles.

As was explained in the last section, ATP synthesis, which happens when protons enter the matrix again, prevents the PMF from building up during OXPHOS. Additionally, complexes I and III produce the majority of the H_2_O_2_ and O^2−^ produced during OXPHOS, which are then scavenged by the antioxidant enzymes in mitochondria [122]. Under healthy circumstances, the proton translocation rate and electron transport rate balance, ensuring the production of enough energy and low levels of ROS [123]. However, keeping the PMF at a high potential causes the membrane to break down, which allows the production of oxidative stress products, including ROS hydrogen peroxide (H_2_O_2_) and superoxide (O^2−^) [123], which can harm cells. A rise in sublethal ROS levels may predispose cells to a better response to oxidative stress, according to a recent study that identified mitochondrial ROS signaling as a mitohormetic route [124].

Additionally, TCA cycle metabolic products such as citrate, a-ketoglutarate, succinate, and fumarate commonly affect epigenetic modifications (nuclear DNA methylation, histone acetylation, and protein hydroxylation and acetylation) [125,126]. Since sirtuins (Sirt) are a direct product of mitochondrial complex I, alterations to NAD^+^ may have an impact on their activity [127,128,129]. Sirt is a signaling molecule that regulates aging-related metabolic and non-metabolic processes [130,131,132]. Additionally, by releasing mtDNA and peptides encoded by mtDNA (such as Humanin, HN, and the mitochondrial open reading frame of the twelve S rRNA-C, MOTS-c), mitochondria modulate the immunological response [133,134]. Mitochondrial collaboration with other cellular devices, such as the internal quality network, can regulate lipid homeostasis, immune response, and cell death [135]. Because maintaining cellular energy homeostasis requires a delicate balance, mitochondria must be better adapted to environmental changes in order to maintain continuous energy production and complete communication with other organelles for cell survival.

### 3.3. Mitochondrial Quality Control

The dynamic nature of mitochondria (fission, fusion, axonal trafficking, biogenesis, and mitophagy) makes mitochondrial homeostasis a critical factor in determining their quality and function [136,137]. It takes a variety of mechanisms, including the monoconidial proteostasis-maintaining mitochondrial unfolded protein response (mtUPR), to respond to malfunctioning mitochondria and ensure organelle maintenance [138], increased mitophagy (a process that eliminates damaged organelles) [139] and biogenesis (a method for producing new mitochondria).

Mitochondrial biogenesis is the cellular process that produces new mitochondria through adding new content, including proteins and membranes, to pre-existing mitochondria. Generally, peroxisome proliferator-activated receptor gamma coactivator 1 alpha (PGC-1α) activates nuclear respiratory factor 1 and 2 (NRF1 and NRF2) to induce mitochondrial biogenesis. Studies have revealed the regulatory role of Sirt1 and adenosine monophosphate-activated protein kinase (AMPK) in mitochondrial biogenesis upstream of PGC-1α [140]. However, there is an apparently impaired mitochondrial biogenesis and reduced mtDNA copy number in neurodegenerative diseases, including AD, that leads to a damaged mitochondrial quantity and quality, and even cell death.

In order to create a more resource-rich organelle, fusion is the biological process that unites many mitochondria into one. Optic atrophy 1 (OPA1), the master regulator of mitochondrial fusion, catalyzes proteolytic processing within mitochondria. When OPA1 is deficient in neurons, it alters the mitochondrial distribution in dendrites and axons, leading to mitochondrial fragmentation and cell-cycle defects [141]. Contrarily, fission helps to promote mitochondrial proliferation by removing unhealthy mitochondria after they split into daughter mitochondria, which are vulnerable to being engulfed by autophagosomes for lysosomal destruction [142]. Human fission protein 1 (hFis1, Fis1p in yeast) and dynamin-like protein 1 (DLP1/Drp1, Dnm1p in yeast) work together to cause mitochondria to split in mammals [143,144,145]. Recently, Drp1 has also been shown to play an essential role in synapse formation.

Notably, the movement of ions and proteins that is necessary for mitochondrial activity is accelerated by the mitochondrial membrane potential (MMP or Δψm). Meanwhile, changes in Δψm and mitochondrial homeostasis crosstalk in AD pathology. Previous studies have indicated that Aβ-induced mitochondrial fragmentation and the loss of membrane potential are associated with the recruitment of Drp1 to mitochondria [146]. However, the lowering of Drp1 in AD mice models enhanced learning and memory abilities, and protected mitochondria from fragmentation and mRNA loss [147]. Additionally, the stabilization/activation of PTEN-induced kinase 1 (PINK1) proteins on the mitochondrial membrane was affected by the defective mitochondrial membrane, which is a hallmark of injured mitochondria [148]. PINK1 firstly phosphorylates and recruits the E3-ubiquitin ligase, Parkin, to mitochondria, and subsequently phosphorylates ubiquitin to feed the Parkin-mediated ubiquitination of mitochondrial outer membrane proteins; this labels and selectively eliminates the damaged mitochondria via the mitophagy pathway [149].

The pathophysiology and development of AD are determined by the presence of defective mitophagy, which causes the buildup of damaged mitochondria [150]. Mitochondrial-containing autophagosomes fuse with lysosomes, which further digest these organelles to form fragments, thus ensuring efficient mitophagy. Recently, neurons from patients with AD have been found to exhibit the abnormal accumulation of autophagosome vacuoles [151]. Not only is Sirt1 implicated in mitochondrial biogenesis, but studies have shown that Sirt1 and Sirt3 also play an important role in the mitophagy of defective mitochondria by highlighting the deacetylation of mitophagy proteins. A recent study found that Sirt1 and Sirt3 were significantly reduced in the cortical regions of the brain in AD patients (leading to the accumulation of defective mitochondria) [152,153]. Furthermore, astrocytes can also help neurons to get rid of defective mitochondria [154]. Mitochondrial quality control determines the disease progression of AD, and several physiological processes for mitochondrial quality control tend to have crosstalk, which may be a potential measure for finding biomarkers and therapeutic targets.

## 4. Potential Targets for AD Therapy

### 4.1. Targeting Mitochondria in AD Therapy

#### 4.1.1. Mitochondrial Division Inhibitor 1 (Mdivi-1)

Mdivi-1, a Drp1 specific inhibitor, inhibited the mitochondrial fission process. Meanwhile, Mdivi-1 was also found to inhibit the activity of the mitochondrial electron transport chain complex I at concentrations (e.g., 50 μM) used to target mitochondrial fission [152].

Mdivi-1 could pass through the BBB. Mdivi-1 was administered orally daily at 10 and 40 mg/kg for 1 month in control and APP/PS1 mice. The Morris water maze test showed that Mdivi-1 could improve learning and memory deficiency, and reduce Aβ plaques in APP/PS1 mice [155]. Mdivi-1, when intraperitoneally injected at 1.5 mg/30 g once every two weeks for three months, improved learning and memory in the Y-maze test, decreased Aβ plaques, increased mitochondrial movement, length, neurite location, and protein content, and increased heme oxygenase-1 protein levels in the hippocampus in CRND8 and wild-type mice [156]. Pretreatment with Mdivi-1 reduced mitochondrial fragmentation in the hippocampus of mice after surgery and in the primary neurons after tumor necrosis factor exposure [157]. However, Mdivi-1 administration’s impact on mitochondrial bioenergetics in animal models still needs to be better understood.

#### 4.1.2. Resveratrol

Resveratrol acts as a natural antioxidant, and can also delay aging and prevent cancer. Resveratrol involves several important signaling pathways, such as AMPK activation, NF-kB signaling inhibition, and antioxidant defenses. Resveratrol decreases neuronal apoptosis, boosts Aβ peptide clearance and the anti-amyloidogenic cleavage of APP, and promotes cholinergic neurotransmission and brain-derived neurotrophic factor (BDNF) synthesis, according to research in experimental AD [158].

Resveratrol facilitates mitochondrial biogenesis and increases the activities of mitochondrial complexes, preserving the mitochondrial membrane potential and thus alleviating mitochondrial dysfunction [159]. These effects of resveratrol may be mediated via the activation of the NAD-dependent protein deacetylase Sirt 1 [160]. Specifically, resveratrol has been found to improve mitochondrial function and biogenesis through the Sirt 1/AMPK/peroxisome PGC1a pathway, which prevents the deleterious effects of oxidative stress [161].

Meanwhile, 26 weeks of treatment with resveratrol in healthy older adults showed that resveratrol had a significant effect on the retention of words over 30 min, which was related to improved glucose metabolism and increased hippocampal functional connectivity [162]. For 15-month-old healthy mice, resveratrol combined with dimethyl fumarate and methylene blue improved short-term and long-term memory [163]. The compounds have been observed to enhance mitochondrial biogenesis, mitophagy, as well as antioxidant defense in the hippocampus by activating the Nrf2/ARE pathway, which reduces oxidative damage to mtDNA [163].

#### 4.1.3. CP2

It has been discovered that the substance CP2 is a tricyclic pyrone molecule that permeates the BBB and builds up in mitochondria [164]. Acyl-CoA: Cholesterol Acyltransferase is inhibited by CP2, which also inhibits mitochondrial ETC complex I activity in a selective and targeted manner [164,165,166]. In vitro studies have demonstrated that CP2 inhibits Aβ aggregation and reduces the cell death caused by Aβ oligomers [167]. Meanwhile, chronic CP2 treatment in vivo has been evaluated in separate cohorts of APP/PS1 or 3×Tg AD-deficient male and female mice. According to research, CP2 reduces amyloid plaques in the brains of APP/PS1 mice and improves cognition in AD models [164,165,166]. Notably, CP2 helps to maintain energy homeostasis (glucose uptake and consumption, glucose tolerance, and insulin resistance), synaptic activity, long-term potentiation, dendritic spine maturation, cognitive function and proteostasis (reduced Aβ and pTau levels), and also decreases inflammation and oxidative stress in the brain and peripheral, ultimately halting the progression of neurodegeneration [164,165,166]. CP2 works on AMPK activation and downstream signaling to increase resistance to oxidative stress, augment mitochondrial bioenergetics, improve glucose uptake and utilization, increase the production of Sirt 1 and 3, reduce glycogen synthase kinase 3 beta (GSK3β) activity, and increase autophagy, levels of BDNF and synaptic proteins in vivo [165,166].

Studies on the bioenergetics of primary cortical neurons have shown that CP2 increases spare respiratory capacity and enhances cellular bioenergetics. This indicates that the mitochondria can generate the energy needed for long-term cell survival and function, even in the face of increased workload or stress [168]. Similar to this, in one study, CP2 increased the AMP/ATP ratio and decreased the basal, ATP-linked, leak-linked, and non-mitochondrial oxygen consumption rates in the brains of APP/PS1 mice [167,168]; this suggested an improved neuronal ETC coupling efficiency, a larger bioenergetic reserve, and a stronger capacity to withstand stress. It was found that 24 h after CP2 was consumed by APP/PS1 rats, there were additional alterations in the metabolic regulation, including an increase in SIRT3, PGC1a, GLUT3, and GLUT4, as well as a decrease in the phospho-PDH/total PDH ratio [169].

### 4.2. Metformin

Metformin, one of the most popular diabetic medications, increases insulin sensitivity and has a number of benefits, such as its ability to improve peripheral glucose absorption and reduce hepatic glucose synthesis [169]. Metformin’s potential therapeutic value in AD has just started to come to light [170,171].

Preclinical studies have shown that tau phosphorylation is associated with insulin resistance and T2D, and is associated with a resistance to clearance and cognitive decline [172]. Metformin has been found to exert neuroprotective effects on the structure and function of the brain in preclinical models and patients with AD in clinical studies. Metformin can improve neuronal survival via the activation of the mTOR pathway in the brain owing to its suppression of tau phosphorylation and cerebral inflammation [173]. In AD animal models, metformin has been shown to reduce amyloid formation, improve memory function and neuronal survival, and decrease neuroinflammation [174]. Additionally, metformin has also been proven to protect endothelial cells [175,176].

To further investigate the specific mechanism of metformin that is involved in the physiology of the brain, an ongoing randomized controlled trial (NCT03733132) has recruited 40 participants undergoing 40 weeks of metformin treatment (>65 years with fasting glucose between 100 and 140 mg/dL and an abdominal girth of >102 cm in men and >88 cm in women) to determine the production of ATP in the brain, the brain structure and blood flow, muscle mitochondrial respiration, as well as cognitive function. In addition, hypoglycemia is regarded as an independent risk factor for dementia [177,178]. There is a risk of iatrogenic hypoglycemia associated with glucose-lowering medications, particularly in elderly T2D patients who have had diabetes for a long time and are on insulin or sulfonylurea [179]. Compared with other antidiabetic drugs, metformin poses a lower risk of hypoglycemia. However, the exact therapeutic effect that has metformin has on dementia has not been fully demonstrated. Most epidemiological studies of people with diabetes have found that metformin use is associated with a lower risk of dementia [180,181] and better cognitive function [182]. Therefore, the repositioning potential of metformin, especially in neurodegenerative diseases, warrants further exploration.

### 4.3. Compounds That Act as “Probiotics”

In order to verify that probiotics have a beneficial impact on the cognitive function of AD patients, a randomized, double-blind, and controlled clinical trial was conducted among 60 AD patients [183]. The patients were randomly divided into two groups (*n* = 30) and treated with either milk or a mixture of probiotics. The probiotic supplementation contained a mixture of *Lactobacillus acidophilus*, *Lactobacillus casei*, *Bifidobacterium bifidum*, and *Lactobacillus fermentum*. After 12 weeks of continuous intervention, compared with the control group, the patients in the probiotic treated group showed a remarked improvement in the mini-mental state examination score, suggesting that probiotic intake has a positively impact on cognitive function.

Recently, in phase III and double-blind trials involving 818 patients with mild AD, investigators used GV-971 (a sodium amin sulfonate) for 4 weeks in order to reshape the intestinal microbiota. It improved the cognitive function, perhaps by decreasing neuroinflammation [184,185,186]. In a small clinical research (10 participants, Clinical Trials.gov Identifier: NCT03856359, Bausch Health and Duke University), the impact of rifaximin, an antibiotic that alters the gut microbiota, on cognition and serum pro-inflammatory markers, as well as the gut microbiota, is currently being investigated. The study aims to determine how dietary changes, probiotic supplements, and neuronal and whole-body energetics may affect the gut microbiota.

### 4.4. Metal Chelation

According to Section 2.2, there may be an accelerated progression of cognitive impairment in AD brains due to poor copper and iron metabolism. According to this, the research found that iron chelation with deferoxamine (125 mg i.m. twice daily/5 days/week for 24 months) significantly slowed the pace at which the ability to perform daily living activities declined in 48 AD patients compared to the placebo group [187]. Deferoxamine enhances the expression of the insulin receptor in the brain and lessens protein oxidation [188]. The deferoxamine treatment of APP/PS1 mice also decreased Aβ and neuronal loss, which was accompanied by the activation of the microglia [189]. In addition, another iron chelator, deferiprone, has been shown to improve cognitive function in a mouse model; moreover, it appears to be associated with tau pathology [190].

Only a small number of metal chelating medications, principally clioquinol (iodochlorhydroxyquin or PBT1) and PBT2, have been studied in clinical trials for the treatment of AD in recent years. Clioquinol works by disrupting the interactions between Aβ and metal ions (copper and zinc), consequently reducing Aβ plaques and oxidative stress. In animal studies, quinoline derivatives have been found to chelate cerebral excesses of iron and copper, and are expected to retard the amyloid plaque progression in humans [191]. However, no clioquinol studies have shown that it is able to significantly improve cognition [192]. Furthermore, the long-term use of quinoline derivatives may result in substantial adverse effects, including mental health issues, which is another important restriction on their therapeutic usage [193]. PBT2 is an analog of 8-hydroxyquinoline that has activity as a copper and zinc translocator, and as a reducing agent for Aβ. PBT2 (30 mg/kg/day) has been found to restore dendritic spine density and reduce Aβ by 13% in pre-clinical testing [194,195]. A phase II trial with 78 patients (50–250 mg/day) revealed its safety, ability to reduce Aβ, and ability to improve cognition [196]. Its safety and tolerability were confirmed in a follow-up phase II trial with 40 participants throughout a 12-month treatment period (250 mg/day); however, there was no appreciable difference in Aβ [197]. In addition, resveratrol, a different chelator, has also received positive reports of encouraging findings [198,199].

## 5. Discussion

A straightforward strategy seems to be glucose supplementation because the bioenergetic system of the brain is affected early in the development of AD and a decline in glucose metabolism and impaired basal metabolism have been widely observed in AD clinical and preclinical trials. However, lower glucose metabolism seems to not relate to decreased circulatory glucose levels and instead, is often associated with increased blood glucose, as in diabetes. Glucose supplementation alone is not a viable strategy for AD treatment. Therefore, enhancing the mitochondrial energy metabolism and improving the impaired brain basal metabolism may be therapeutic strategies. In addition, the targeted normalization of proper mitophagy is believed to be a potent therapeutic design for these diseases. Interestingly, the inhibition of mitochondrial respiration in many cases extends lifespan, probably due to the reduced mitochondrial production of reactive species or an adaptive mitotic response to selective stress [200,201,202,203,204,205,206,207,208]. For instance, blocking ETC complex I lengthens life expectancy in non-mammalian animals [209], and several compounds that are beneficial in animal models of AD have been found to have mild complex I inhibitory activity. When mitochondria utilize lipids as a fuel source for ketone bodies, the increased mitochondrial function may accelerate the loss of white matter in the brain, as demonstrated in female animal models of aging and AD; human brain imaging in women also confirmed this phenomenon. Furthermore, increased mitochondrial respiration in microglia may contribute to oxidative stress damage.

Therapeutics that target the systemic metabolism and mitochondria in order to enhance the bioenergetic system capacity and mitochondrial function may be able to prevent, postpone, and reverse the negative effects of this important AD driver; however, systemic metabolism and mitochondrial-targeted therapy are difficult due to AD’s complicated and interlocking processes, the disease’s heterologous genetic and environmental causes, and these factors.

## Data Availability

Not applicable.
